# Supplementary material for: A Comprehensive Assessment of Ultraviolet-Radiation-Induced Mutations in Flammulina filiformis Using Whole-Genome Resequencing
Source: J Fungi (Basel). 2024 Mar 20;10(3):228. doi: 10.3390/jof10030228 (PMC10971301; doi:10.3390/jof10030228)
Supplement: Supplementary file 1 [file jof-10-00228-s001.zip › Supplementary Material S8/KEGG annotation/out/64381550635650.os/KO/out_map/map04918.html]

KEGG PATHWAY: Thyroid hormone synthesis - Reference pathway


|  |  |
| --- | --- |
| **Thyroid hormone synthesis - Reference pathway** |  |

[
Pathway menu
| Organism menu
| Pathway entry
| Show description
| User data mapping
]

Thyroid hormones triiodothyronine (T3) and thyroxine (T4) are essential for normal development, growth and metabolic homeostasis in all vertebrates, and synthesized in the thyroid gland. The functional unit of the thyroid gland is the follicle, delimited by a monolayer of thyrocytes. Polarized thyrocytes surround the follicular lumen; with their basal and apical surfaces facing the bloodstream and the lumen, respectively. To synthesize thyroid hormones, thyrocytes take up iodide at their basal side and concentrate it into the lumen. They also secrete in this lumen the specialized protein thyroglobulin (TG) which serves as a store for the hormones. In the follicular lumen oxidation of iodine, iodination of tyrosines (MIT, 3-monoiodotyrosine; DIT, 3,5-diiodotyrosine) and coupling of iodotyrosines takes place on tyrosine residues in TG, resulting in T3 and T4 synthesis. Iodinated TG is resorbed through the apical membrane and degraded to form T3/T4 in lysosomes; the T3/T4 is then secreted through the basal membrane.

|  |  |
| --- | --- |
| Reference pathway | 100% |
